# Rapid Fluorescent Probe Detection of Magnesium Impurities in High-Purity Lithium Carbonate Brine Systems

**DOI:** 10.3390/molecules30040776

**Published:** 2025-02-07

**Authors:** Yan Li, Huaigang Cheng, Yueyue He, Jing Zhao

**Affiliations:** 1 Institute of Resources and Environmental Engineering, School of Environment and Resources, Shanxi University, Taiyuan 030006, China; 18856123825@163.com (Y.L.); 20241021@llu.edu.cn (Y.H.); sxzj1112@163.com (J.Z.); 2 Salt Lake Chemical Engineering Research Complex, Qinghai University, Xining 810016, China; 3 Department of Chemical and Materials Engineering, Lvliang University, Lvliang 033000, China

**Keywords:** fluorescent probe, high-purity lithium carbonate, magnesium impurity, water solubility, solvation effect

## Abstract

The magnesium impurities in lithium carbonate cannot be detected quickly in an aqueous environment. To solve this bottleneck problem, this study proposes a new method for the rapid detection of trace Mg^2+^ in lithium carbonate using a water-soluble fluorescent probe. A water-soluble fluorescent probe A was obtained by introducing hydroxyl groups on a fluorescent oxazole ring. After modification, the hydrogen bonding between the probe and water molecules increased by more than 62 times. Consequently, the energy loss of outward transfer of the fluorescent probe increased, resulting in weak fluorescence in saline systems. Mg^2+^ was captured by N on the oxazole ring and O on the phenolic hydroxyl group through a 1:1 coordination ratio within the probe structure. The hydrogen bonding attraction between the complex and water molecules increased 16 times. Additionally, the orbital energy gap was reduced from 2.817 to 0.383 eV. Meanwhile, the Mg^2+^ impeded the phototropic electron transfer effect process, resulting in enhanced fluorescence and completing this process within 3 to 10 s, with a detection limit of 6.06 μmol/L. This method can promote the real-time and rapid quality control of Mg^2+^ impurities in the refining and purification of lithium carbonate, as well as effectively reduce production costs.

## 1. Introduction

High-purity lithium carbonate is a crucial raw material due to its distinct advantages [1], especially in the new energy industry, as an important support for lithium-ion battery materials [2,3]. Salt lake lithium carbonate is a vital source of this material. However, the lithium carbonate preparation from salt lakes is susceptible to interference from Mg^2+^ impurities since it is difficult to separate magnesium and lithium in salt lake brine [2]. The purity of lithium carbonate must exceed 99.5% in the positive electrode material of lithium batteries [1]. The product quality is significantly influenced by the presence of Mg^2+^ impurities, which impairs the battery’s performance and lifespan. Salt lake lithium carbonate is widely used in the wet process to prepare high-purity lithium carbonate; therefore, the real-time monitoring of water-soluble salt components is crucial for process regulation and product quality enhancement [3]. The current challenge in producing high-purity lithium carbonate is the rapid detection of Mg^2+^ impurities, which has become a bottleneck in lithium carbonate purification [4,5]. Thus, developing an efficient detection method for Mg^2+^ impurities can improve the lithium carbonate purity, reduce costs, and enhance product quality.

The most recent advancements in Mg^2+^ detection methods are based on different techniques, such as inductively coupled plasma chromatography [6,7], spectroscopy [8,9], nuclear magnetic resonance, spectrophotometry, and voltammetry [10]. These methods exhibit satisfactory detection limits and a wider linear range [11]. However, they often necessitate static sampling, cumbersome and complex pre-processing procedures [12], generally need longer detection times, and are unsuitable for online detection. Hence, there are great difficulties in the precise regulation of high-purity lithium carbonate processes in practical applications [13]. Laser-induced fluorescence detection technology is widely used in the chemical processing of metal ions in the fluid dynamic changes due to its in situ non-contact, high detection accuracy, and real-time response [14,15,16].

The synthesis of specific fluorescent probes is a crucial step in laser-induced fluorescence [17]. To successfully detect Mg^2+^ impurities in the wet refining of lithium carbonate, the probe must possess Mg^2+^ fluorescence responsiveness and water solubility. Many advances have been made in designing fluorescence-responsive probes for Mg^2+^ detection. For example, researchers have synthesized the Schiff base fluorescent probe (H_2_L) and employed it for the detection of Mg^2+^ in a 1:4 H_2_O/DMSO solution [18]. A study prepared nitrogen-doped carbon dots (NCDs) with enhanced ratiometric photoluminescence (PL) for detecting Mg^2+^ in cells [19]. However, the PL intensity of NCDs was pH-dependent. A novel solvent-directed Schiff base was synthesized, which contained multiple corresponding fluorescent probes L. The fluorescence properties of L demonstrated a significant solvent effect, requiring sufficient dilution with acetonitrile (1:9v/v) for Mg^2+^ detection in tap water [20]. Silver nanoparticles (AgNPs) [21] were synthesized for the quantitative detection of Mg^2+^ in water samples, but their selectivity was determined by the pH value. Other Mg^2+^ fluorescent probes were synthesized based on Schiff bases [22,23], including 1,8-naphthalene dicarboximide derivatives [24]. The fluorescence response of these probes was dependent on the organic phase, and samples were pre-treated by dissolving the organic phase. Regrettably, there is a lack of research on water-soluble Mg^2+^ probes. Therefore, the online monitoring of high-purity lithium carbonate remains elusive via the wet purification method. The correlation between the probe response to Mg^2+^ and its water solubility, attributed to the solvation effect, suggests that addressing the solvation effect solves the challenges associated with Mg^2+^ detection in brine systems. Nevertheless, only a limited number of studies have reported the impact of the solvent on the fluorescence response, particularly solvation effects, the role of electrostatic forces, mutual polarization, and hydrogen bonding [25,26]. Therefore, the solvation effect represents a crucial avenue for addressing water solubility and Mg^2+^ fluorescence responsiveness in a unified manner.

To address the issues above, this study investigates the solvation effect on the functional group of fluorescent-group-directed modification. The polarity of the probe is altered by grafting hydrophilic groups to modify the solvent (water molecules) and solute (probe A) by tuning the hydrogen bonding interactions. Furthermore, the detection mechanism is investigated in the brine system, and the solvation effect on the fluorescence signal is analyzed. The primary objective is to develop a water-soluble fluorescent probe and a new detection method for Mg^2+^ impurity detection. The proposed method is expected to overcome the current limitation of the probe in the brine system, which cannot produce a fluorescence response after capturing free Mg^2+^. This study will provide a rapid online detection of Mg^2+^ impurities in the lithium carbonate refining process.

The detection reagent shows weak fluorescence due to the synergistic effect of the solvation and the phototropic electron transfer (PET) effects in saline. The changes in interaction energy, electron density, and orbital energy gap result in fluorescence enhancement after capturing Mg^2+^, mainly due to the ligand-to-metal electron transfer process and lower reduction potential of Mg^2+^ in water. These processes block the PET process in the probe structure, thus showing fluorescence enhancement.

## 2. Results and Discussion

### 2.1. Synthesis of Assay Reagents

**Figure 1:**
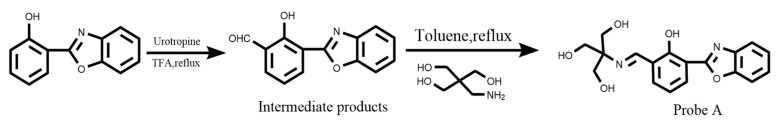
Synthesis route of fluorescent probe A.

First, a formylation reaction was carried out to increase the reactivity of 2-(2 hydroxyphenyl) benzoxazole, dissolving 2-(2-hydroxyphenyl) benzoxazole in trifluoroacetic acid. Then, hexamethylenetetramine was added to the above solution, forming a yellow solid with a productivity of 64.3%. ^1^H NMR (600 MHz, DMSO-d_6_) δ 10.46 (s, 1H), 8.32 (d, *J* = 7.7 Hz, 1H), 7.93 (d, *J* = 7.7 Hz, 1H), 7.49 (dq, *J* = 15.6, 7.9 Hz, 4H), 7.24 (t, *J* = 7.7 Hz, 1H). Further modification was performed by grafting hydrophilic groups. The synthesized compound was dissolved in toluene, and tris(hydroxy)aminomethane was added. A yellow solid was obtained after reaction completion, labeled as fluorescent probe A; the synthesis route is shown in Figure 1 (the solid diagram of the electrostatic potential can be referred from Figure S1). ^1^H NMR (600 MHz, DMSO-d_6_) δ 15.58 (s, 1H), 8.60 (s, 1H), 8.11 (dd, *J* = 7.5, 1.9 Hz, 1H), 7.75–7.72 (m, 2H), 7.62 (dd, *J* = 7.8, 1.9 Hz, 1H), 7.38–7.34 (m, 2H), 7.18–7.12 (m, 1H), 6.68 (t, *J* = 7.6 Hz, 1H), 5.07 (s, 2H), 4.36 (s, 6H). The high-resolution mass spectra at proton number/charge ESI-MS m/z: [M + H]^+^ 343.129 exhibited a strong molecular ion peak attributed to [Probe A]^+^. The theoretical molecular weight of the product was 342.12. The configuration method of the detection reagent was as follows: take 6.847 mg of probe A and dissolve it in 10 mL of N, N-dimethylformamide (DMF) to prepare a detection reagent with a concentration of 2 × 10^−3^ mol/L.

### 2.2. Optical Properties of Detection Reagents in Brine System

To study the optical properties of the detection in the brine system, the microscopic interactions between water molecules and probe A, the fluorescence properties of detection reagents in different solvents, and the fluorescence stability in salt water were investigated.

**Figure 2:**
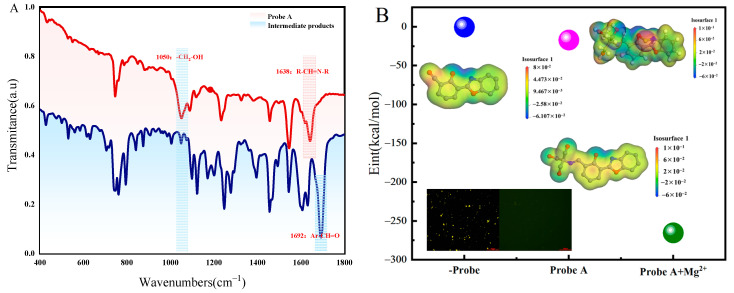
Properties before and after modification. (**A**) Local infrared spectra of intermediate and final product probe A (Figure S2. Complete infrared spectrogram). (**B**) The interaction energy between intermediate products, probe A, and water molecules (As shown in the colored balls); the electrostatic potential of intermediate products, probe A, and probe A complexed with Mg^2+^; fluorescence microscopy images of intermediate products and detection reagents (Figures S9 and S10).

First, the hydrophilicity of the probe was investigated by testing functional groups, microscopic hydrogen bonding, and the dispersion effect. Figure 2A shows that a strong absorption peak of Ar-CH=O was observed at 1692 cm^−1^, influenced by the benzene ring substituent, and the spacing effect of the reaction intermediate disappeared. The final product revealed -CH_2_-OH and R-CH=N-R vibration peaks at 1050 cm^−1^ and 1638 cm^−1^, respectively, indicating the attachment of the hydrophilic group on the fluorophore. Second, the electrostatic potential of the modified substance and its interaction energy with water molecules were analyzed. Figure 2B illustrates that the polarity of the probe increased due to the increased negatively charged areas on the phenolic hydroxyl group and oxazole ring, facilitating hydrogen bond formation with water molecules [27]. The interaction energies with water molecules before and after modification were −0.271 and −16.949 kcal/mol, respectively, suggesting stronger hydrogen bonding attraction [28,29]. Finally, the dispersion effect before and after modification was observed in the brine system. As shown in Figure 2, the substance was uniformly distributed in brine solution before modification, and after modification, the probe was completely miscible with water. The hydrophilic groups were successfully implanted into the fluorophore, resulting in enhanced hydrogen bonding between the modified probe A and water molecules.

**Figure 3:**
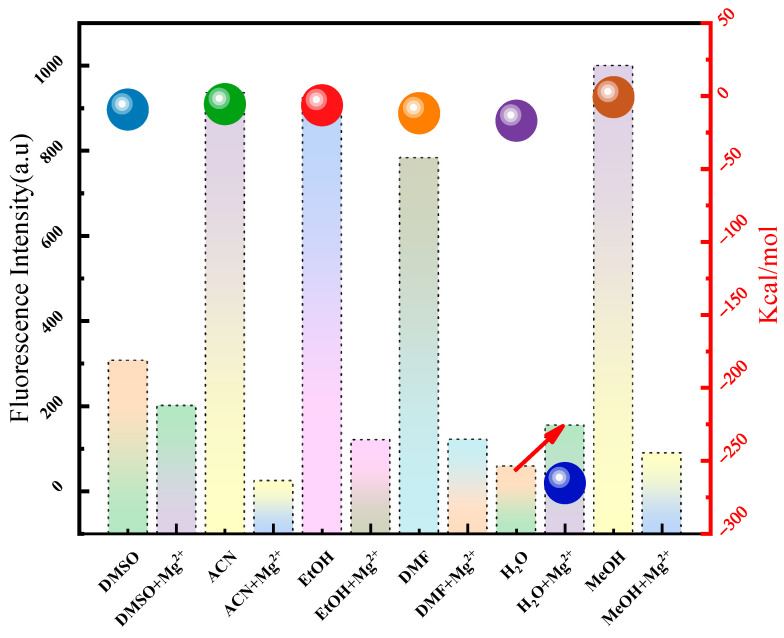
The maximum fluorescence intensity of the detection reagent in different solvents and the interaction energy between probe A and molecules in different solvents (Colored balls indicate the magnitude of the interaction energy, the red arrow indicates that the fluorescence intensity increases only in aqueous solution). (Figure S3. Fluorescence spectra of detection reagents before and after identifying Mg^2+^ in different solvents. Table S1. Detailed parameters in the interaction energy calculation process).

The difference between the fluorescence properties of the probe in the brine system and other solvents was analyzed via the fluorescence response and microscopic interaction energy. First, the differences in fluorescence signals of the detection reagent were analyzed in different solvents (solvent type: DMSO, CAN, EtOH, DMF, H_2_O, MeOH). Sample preparation details during the detection process: Take 10 μL of the detection reagent and add it to 3 mL of various solvents. Scan the fluorescence signals of the above samples using a fluorescence spectrometer. The parameter settings of the fluorescence spectrometer are shown in the Materials and Methods section below. As shown in the bar chart in Figure 3, the strongest fluorescence signal was observed in methanol, and the weakest response was observed in the brine system. The fluorescence intensity of the brine system was 1/17 of that of methanol. The fluorescence intensity of the probe decreased with the increase in solvent polarity in different solvents. The interaction energy between probe A and different solvents was also analyzed, as shown by the sphere in Figure 3. The interaction energy of probe A was largest with water molecules and smallest with methanol molecules: 1.8–20 times higher in water than those with other solvent molecules. These results reveal that probe A showed a weak fluorescence in the solvent with strong interactions. Moreover, it showed a strong fluorescence signal when the interaction was weak, indicating that different interactions between solvent molecules and probes directly impact the fluorescence response [30,31,32,33]. The fluorescence signal of the probe was also affected by the relaxation time of the solvent molecules and the average lifetime of the probe’s excited state [34,35,36].

**Figure 4:**
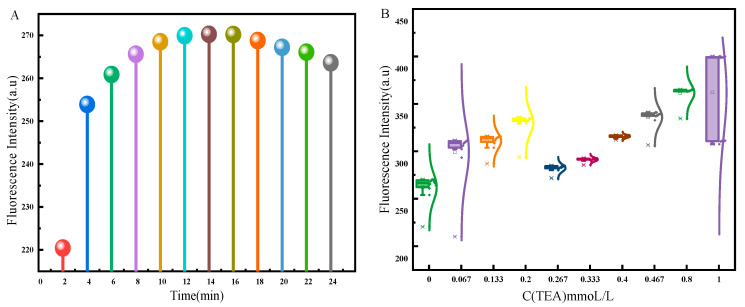
The changes in fluorescence intensity of detection reagents over time under different conditions: (**A**) the maximum fluorescence intensity of detection reagents at different times in physiological saline (the 2D fluorescence spectral profile of the detection reagents over time is shown in Figure S4); (**B**) the box plot of the maximum fluorescence intensity of detection reagents over time in physiological saline with different concentrations of trimethylamine added (the size of the box plot reflects the concentration of fluorescence intensity under a certain condition and thus reflects the stability of fluorescence intensity, or you can refer to Figure S6).

Fluorescence stability is one of the most important parameters for accurate detection [37,38]; therefore, the stability of the probe was investigated in saline. An amount of 10 μL of probe detection reagent with a concentration of 2 × 10^−3^ mol/L was taken and added to 3 mL of deionized water. The fluorescence spectrum of the mixed sample was scanned every 2 min and the detection was repeated 12 times. The results are shown in Figure 4A. The changing fluorescence intensity with time indicated less stability of the fluorescence signal under these conditions, which could interfere with the accurate assessment of the impurity ion during the detection process (Figure 4A). Different amounts of triethylamine were added to the above-mixed solution. The volume of triethylamine was 0, 10, 20, 30, 40, 50, 60, 70, 120, and 150 μL, and the concentration of triethylamine was 2 × 10^−2^ mol/L. The results are shown in Figure 4B. When the triethylamine content was 0.334 × 10^−3^ mol/L (the volume of triethylamine added was 50 μL), the box shape was shortest, indicating the strongest fluorescence stability. The change in fluorescence spectra under this condition is shown in Figure S7. These results suggest that triethylamine played a positive role in photostability by lowering the dielectric constant of the solvent [39]. By adding a known amount of triethylamine to the detection system, its fluorescence signal stability was maintained in the brine system, realizing accurate detection results.

The polarity of fluorescent probe A increased after modification and was demonstrated by functional group and hydrogen bonding interactions. The probe’s fluorescence signals in different solvents were analyzed through the microscopic interaction energy, suggesting that the increased polarity after modification resulted in a larger interaction energy with water molecules. Therefore, a weaker fluorescence intensity was observed in the brine system, which highlighted the increase in fluorescence during detection with better fluorescence stability. These results verified the feasibility of the detection probe in the brine system.

### 2.3. Detection Sensitivity for Mg^2+^ in Brine System

To investigate the probe’s sensitivity to Mg^2+^ detection in the brine system, the detection limit and detection response were investigated. Concentration titration and response time experiments were carried out to determine Mg^2+^ in the brine system.

**Figure 5:**
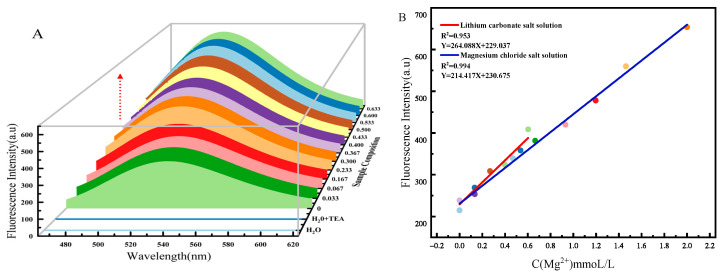
Detection ability of detection reagents for different concentrations of Mg^2+^: (**A**) Fluorescence spectra of detecting different concentrations of Mg^2+^ in saline solution (Red arrows indicate the trend of increasing fluorescence intensity with increasing Mg^2+^ concentration, the 2D fluorescence spectral profile is shown in Figure S4). (**B**) The linear relationship between Mg^2+^ concentration and fluorescence intensity during the detection process.

The sensitivity of the probe to determining Mg^2+^ concentration in saline was evaluated by fluorescence titration experiments. An amount of 10 μL of detection reagent was transferred into centrifuge tubes, and then different volumes of Mg^2+^ salt solution were added to the centrifuge tubes, and the fluorescence emission spectra of the scanned samples are shown in Figure 5A (the final Mg^2+^ concentrations in the test sample were 0, 0.033, 0.067, 0.167, 0.233, 0.300, 0.367, 0.400, 0.433, 0.500, 0.533, 0.600, and 0.633 mmol/L). Figure 5A illustrates that the fluorescence intensity increased linearly with increasing Mg^2+^ concentration, and earlier studies suggest that this phenomenon may also be influenced by solvent polarity and dielectric constant [40]. The relationship between Mg^2+^ concentration and the fluorescence intensity obtained from the assay was fitted. The R^2^ value of 0.994 revealed a good linear relationship between the fluorescence intensity and Mg^2+^ concentration [41]; the corresponding fluorescence spectrogram for this working curve is shown in Figure S8. Considering the Lambert–Beer law (the quantitative relationship *I*_f_ = *φI*_o_(1−e^−2.3*εbc*^) between the fluorescence intensity *I*_f_, the fluorescent substance concentration C(mol/L), and the stimulated luminescence *I*_0_ [*φ*—fluorescence quantum yield; *ε*—molar absorptivity, L/(mol·cm); and *b*—thickness of the liquid pool, cm]), the correlation between Mg^2+^ concentration and fluorescence intensity in lithium carbonate solution was calculated, as shown in Figure 5B (red curve). The slope of the curve (K) was 264.088 L/mmol, and further calculation shows that the detection limit of Mg^2+^ impurities in high-purity lithium carbonate was 6.06 µmol/L [42,43,44] (relevant calculated data on detection limits are shown in Table S2). Five sets of high-purity lithium carbonate solutions were configured with known Mg^2+^ impurity concentrations (Mg^2+^ concentration: 0, 0.067, 0.201, 0.335, 0.402 mmol/L). An amount of 3 mL of lithium carbonate solutions was prepared with different Mg^2+^ concentrations and was placed in 5 mL centrifuge tubes. An amount of 10 μL of detection reagent was added to obtain the test samples. The fluorescence spectra of the test samples were scanned and fluorescence peaks were obtained at 234.308, 250.271, 281.265, 312.089, and 327.501 a.u., respectively. The measured fluorescence values were substituted into the working curve for calculation. The detection values of Mg^2+^ were 0.019, 0.08, 0.197, 0.314, and 0.372 mmol/L, and further, the average relative error of the five experiments was calculated 0.017. The test reagent exhibited a good sensitivity to Mg^2+^ concentration in the saline solution.

**Figure 6:**
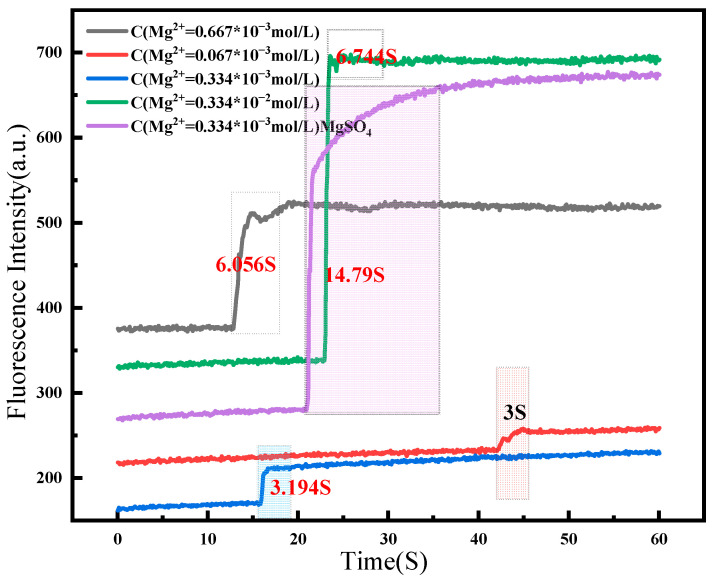
Detection of the fluorescence response time of Mg^2+^ at different concentrations/Mg^2+^ in different solutions.

The response time indicates the detection sensitivity, and the change in fluorescence intensity with time was analyzed when the probe detected different concentrations of Mg^2+^ in different systems. The fluorescence intensity was plotted at the peak of the detection system’s fluorescence spectrum versus time (Figure 6). The detection time increased with Mg^2+^ concentration in the detection solution; however, there was no large gap, and it was completed within 3–10 s (Figure 6). The detection time increased with the Mg^2+^ concentration. To assess the time required for detection, the change in fluorescence intensity with time was scanned for the Mg^2+^ detection in lithium carbonate solution (Figure 6); the assay took only 14.790 s to detect Mg^2+^, confirming the fast response of the probe. Titration and response time experiments investigated the probes’ sensitivity to different concentrations of Mg^2+^ in saline and the speed of detection.

### 2.4. Specific Detection of Mg^2+^ in Saline Solution

Whether the fluorescent probe can specifically recognize the ions directly affects the accuracy of the detection results. Therefore, the specific recognition of Mg^2+^ was investigated using selectivity and interference experiments.

**Figure 7:**
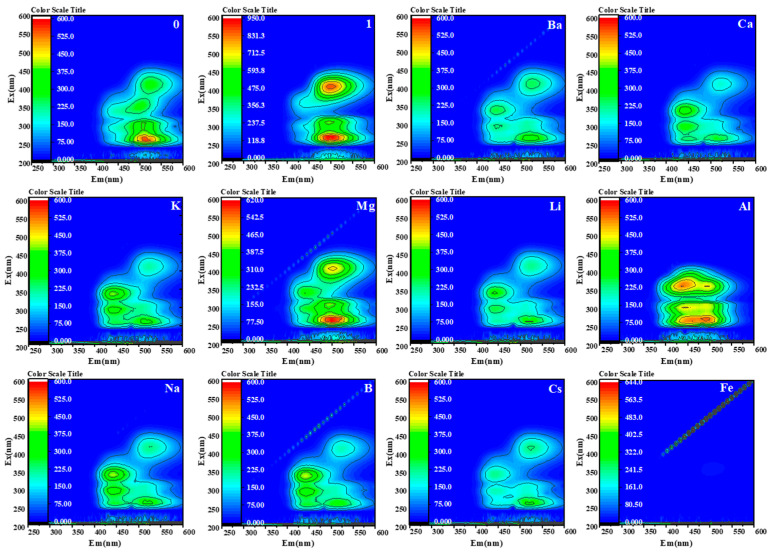
Contour fluorescence spectra of different ions in saline detected by detection reagents (standardizing the three-dimensional fluorescence data, subtracting the fluorescence value of the blank, and eliminating Rayleigh scattering to obtain the contour fluorescence spectrum shown in Figure 7). Figure 7(0) shows the contour fluorescence spectrum of the detection reagent when there are no ions in the detection system. Figure 7(1) shows the outline fluorescence spectrum when the measured ion in the detection system is Mg^2+^ and the concentration is 1 mmol/L).

**Figure 8:**
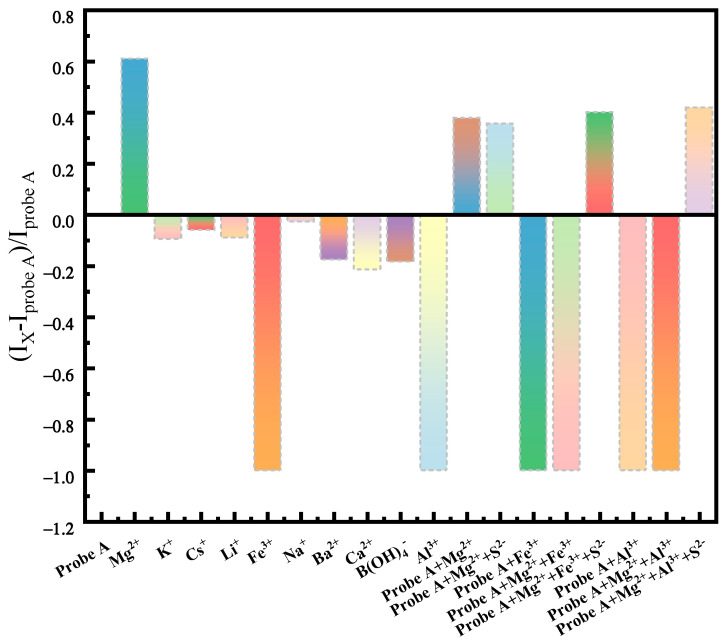
Fluorescence enhancement factor (FEF) in the presence of various metal ions with Mg^2+^ (S^2−^ in the figure represents the main component responsible for precipitation in the masking agent Na_2_S).

Selectivity experiments were conducted. The contour fluorescence spectra of assay reagents were compared and analyzed for the detection of different ions (ionic species Ba^2+^, Ca^2+^, K^+^, Li^+^, Al^3+^, Mg^2+^, Na^+^, B(OH)_4_^−^, Cs^+^, and Fe^3+^ (the concentration of each ion was 0.667 mmol/L). As shown in Figure 7, under full-wavelength excitation and emission, this method only has strong luminescent centers in the contour fluorescence spectrum when detecting Mg^2+^, and no luminescent centers when detecting other ions, or weak fluorescence intensity in the emission wavelength range of 480~530 nm and excitation wavelength ranges of 250~270 nm and 390~410 nm. Further, the values of the fluorescence enhancement factor were analyzed before and after adding metal ions (FEF = (Ix − Iprobe A)/IProbe A), and the results are shown in Figure 8. The detection probe showed a fluorescence-enhanced response only to Mg^2+^. These results were in agreement with contour fluorescence spectra graphs of the different ions recognized by the detection probe, validating that the probe could recognize Mg^2+^ specifically [45].

An interferometric experiment was carried out to determine the interference of different metal ions with the probe’s detection response. The fluorescence intensity did not change significantly in the presence of Li^+^, Na^+^, K^+^, Ca^2+^, Zn^2+^, Fe^3+^, Al^3+^, and Mg^2+^ (Figure 8). When Al^3+^, Fe^3+^, and Mg^2+^ were present, a fluorescence burst occurred. The effect of Al^3+^ and Fe^3+^ on the detection process was removed by adding the masking agent Na_2_S (Figure 8). According to Figure 8, when the horizontal axis is Probe A + Mg^2+^\Probe A + Mg^2+^ + S^2−^, the corresponding vertical axis FEF shows values of 0.379 and 0.357, indicating that the masking agent will not interfere with the fluorescence response of Mg^2+^. When Mg^2+^ coexists with Fe^3+^ and Al^3+^, a masking agent is added, as shown in Figure 8; when the X-axis is Probe A + Mg^2+^ + Fe^3+^\Probe A + Mg^2+^ + Fe^3+^ + S^2−^, the Y-axis FEF changes from −1 to 0.402, and when it is Probe A + Mg^2+^ + Al^3+^\Probe A + Mg^2+^ + Al^3+^ + S^2−^, the vertical axis FEF changes from −1 to 0.42, which is not significantly different from the FEF when only Mg^2+^ is present. These results suggest that adding Na_2_S could effectively remove the influence of Fe^3+^ and Al^3+^ on the detection process. Moreover, the detection probe exhibited a better anti-interference ability for Mg^2+^ detection in the brine solution.

Selectivity and interference experiments suggested that the detection probe could specifically identify Mg^2+^. In the actual detection process, some metal ions did not affect the detection process. The masking agent Na_2_S eliminated the effect of Al^3+^ and Fe^3+^. Overall, the detection probe showed a better detection ability for Mg^2+^, specifically in the detection process.

### 2.5. Detection Mechanisms

The detection mechanism of Mg^2+^ by fluorescent probe A was investigated from the coordination relationship and recognition site. Then, the possible reasons for the change in apparent fluorescence signals were explored from the changes in electron distribution, orbital energy gap, and microscopic interaction energy.

**Figure 9:**
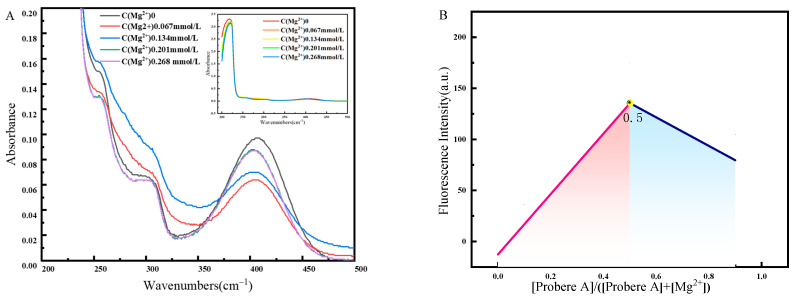
Structural changes before and after probe A recognizes Mg^2+^: (**A**) UV absorption spectrum changes before and after probe A chelates Mg^2+^. (**B**) Job’s working curve for Mg^2+^ detection.

To investigate the mechanism of Mg^2+^ detection, the ligand relationship between fluorescent probe A and Mg^2+^ was first analyzed. Figure 9A shows that the equal absorption points in the UV absorption spectrum after adding Mg^2+^ reflected that probe A reacted with Mg^2+^ to form new substances [46]. The absorption spectra before and after probe complexation with Mg^2+^ were the same, indicating that the fluorescence change of the probe after complexation with Mg^2+^ was attributed to the excited state phenomenon [22]. Further, the Job’s working curves before and after recognition revealed a clear inflection point at 0.5 (Figure 9B). The peak at m/z 365.11 in high-resolution mass spectrometry was attributed to [probe A + Mg^2+^]^+^ (this data source can be viewed in Figure S11), indicating that Probe A formed a complex with Mg^2+^ at a 1:1 stoichiometric ratio. Finally, the NMR hydrogen spectrum of the complex was used to investigate the recognition site. The nuclear magnetic resonance (NMR) hydrogen spectrum experiment was conducted on the complex product, and the ^1^NMR hydrogen spectrum data indicate that Ph-CH=N contained -CH at *δ* = 3.48 (this data source can be viewed in Figure S12), suggesting that the C=N double bond on the oxazole ring was broken and N participated in the coordination reaction to form -C-N-Mg-.

**Figure 10:**
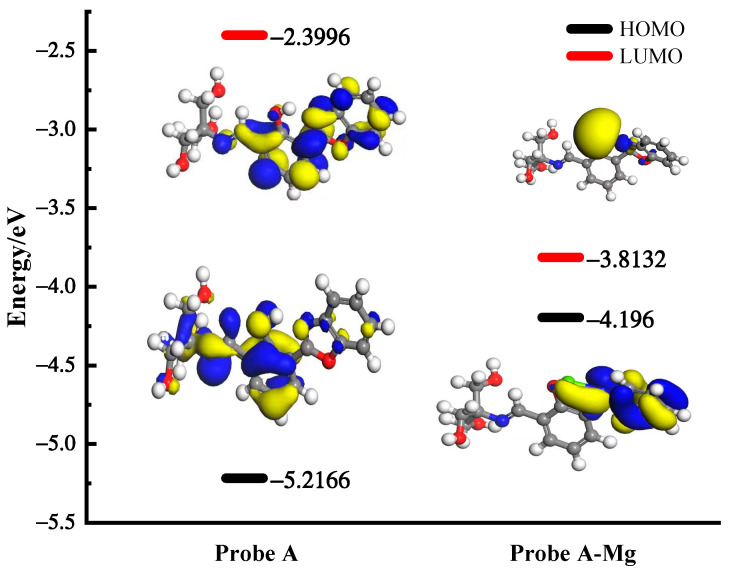
Changes in molecular orbital energy gap before and after probe A chelates with Mg^2+^ (detailed data are presented in Table S3).

To explain the principle of apparent changes in the fluorescence signal, the first step was to analyze the changes in electron density before and after recognition. Figure 10 reveals that the electrons were mainly concentrated on phenolic hydroxyl groups and the oxazole ring before unliganding. This confirms that the structure exhibited fluorescence emission and a strong electron-donating ability [23]. Under light excitation, PET [47] occurred since the electrons located in the highest occupied molecular orbitals (HOMOs) jumped to the lowest unoccupied molecular orbitals (LUMOs), resulting in a weak fluorescence [48,49]. Further, the molecular orbital energy gap changes were analyzed before and after complexation (Figure 10). The energy gap between HOMO-LUMO in the probe A structure before incoordination was 2.817 eV. The energy gap between HOMO-LUMO of the probe A + Mg^2+^ complex was 0.383 eV. The smaller energy contributed to the stronger fluorescence at excitation wavelengths [42], which agreed with the detection results presented in Figure 5A. After capturing Mg^2+^ by the N atom on the oxazole ring and O atom on the phenolic hydroxyl group, the electrons were transferred from benzoxazole and concentrated around Mg^2+^, suggesting a ligand-to-metal electron transfer [23].

### 2.6. Discussion

**Figure 11:**
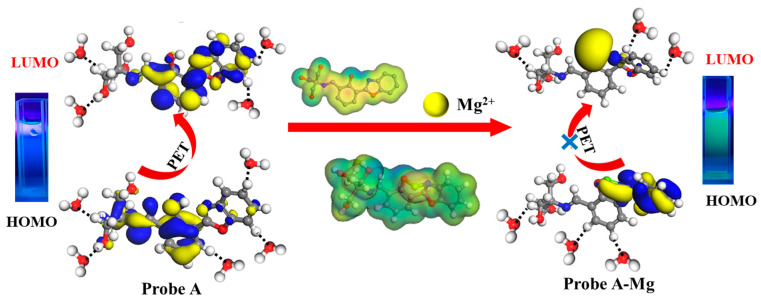
Schematic diagram of the test procedure.

In summary, the polarity of probe A increased after hydrophilic modification before capturing Mg^2+^. The interaction energy with water molecules was about 62 times that before modification (Figure 2B). The stronger hydrogen bond attraction reduced the excited state lifetimes of probe A in water molecules. The rate of excited state molecule jumping was accelerated [50,51,52], the loss of externally transferred energy from probe A increased, the oxazole ring emitted fluorescence, and a strong electron-donating ability resulted in a weak fluorescence before capturing Mg^2+^. Figure 2 indicates that the polarity of the complex increased after capturing Mg^2+^ by probe A, and the interaction with the water molecules was 16 times stronger. Moreover, the complex showed weak fluorescence; however, the Mg^2+^ in the complex hindered the PET process on the benzoxazole ring [53]. Polar water molecules lowered the reduction potential of Mg^2+^ and also contributed to blocking the PET process. This double effect enhanced fluorescence, as shown in Figure 11. The fluorescence enhancement signal detected followed the Lambert–Beer law between the fluorescence intensity and the fluorescent substances concentration. The increase in Mg^2+^ concentration resulted in an enhanced fluorescence response. Therefore, this probe can be used to quantitatively detect trace amounts of Mg^2+^ impurities in lithium carbonate solutions.

## 3. Materials and Methods

### 3.1. Materials

Reagents including 2-(2hydroxyphenyl) benzoxazole (98%), trifluoroacetic acid, hexamethylenetetramine, triethylamine (99%), N,N-dimethylformamide, dimethyl sulfoxide-d6 (99.9%), deuterochloroform-d (D, 99.8% + 0.03% V/V), tris(hydroxy)aminomethane (super pure), ethanol absolute, The above reagents were purchased from Shanghai McLin Biochemical Technology Co., Ltd., Shanghai, China. Magnesium sulfate, MgCl_2_·6H_2_O, Li_2_CO_3_, calcium chloride anhydrous, anhydrous borax, CaCl_2_, LiCl·6H_2_O, KCl, FeCl_3_·6H_2_O, NaCl, CsCl, AlCl_3_, BaCl_2_·2H_2_O, and Na_2_B_4_O_7_·10H_2_O were analytically pure reagents, The above reagents were purchased from Tianjin Fengchuan Chemical Reagent Technology Co., Ltd., Tianjin, China.

Instrumentation included a UV-Vis spectrophotometer (UV-26001, Agilent Shanghai Co., Ltd., Shanghai, China), nuclear magnetic resonance spectrometer (NMRS) (AVANCE III HD 600 MHz, Bruker GmbH, Ettlingen, Germany), high-resolution liquid/mass spectrometer (Thermo Scientific Q Exactive, Thermo Fisher Scientific, Waltham, MA, USA), Agilent Fluorescence Spectrophotometer (S6 JAGUAR, Cary Eclipse, Agilent, Santa Clara, CA, USA), and Fourier-Transform Infrared Spectrometer (NVENIO-S, Brooke, Hamburg, Germany).

### 3.2. Methods

**Synthesis method for probe A.** The following reagents were weighed and subsequently reacted at 80 °C for 5 h: 0.844 g of 2-(2-hydroxyphenyl)benzoxazole, 20 mL of trifluoroacetic acid, and 2.800 g of hexamethylenetetramine. Subsequently, 0.238 g of the reaction product of the first step (0.146 g of tris(hydroxy)aminomethane in 10 mL of toluene) should be weighed, and the mixture should be reacted for 6 h at 110 °C. The change in electrostatic potential of each substance during the reaction is shown schematically in Figure S1 in the supporting information.

**Procedure for UV Absorption Spectra of Probe A Before and After Complexation with Mg^2+^.** A UV-26001 spectrophotometer was used to scan the changes in the UV absorption spectra of probe A before and after complexation with Mg^2+^. The testing method comprised a preheating phase of the instrument for a duration of 30 min, followed by the configuration of the experimental parameters. These parameters included a wavelength range of 200–800 nm, a high scanning speed, a sampling interval of 0.5, a single scanning mode, a slit width of 1.0, and an integration time of 0.1 s. The sample should be prepared by taking 10 μL of the detection reagent and dissolving it in 3 mL of deionized water. Then, 10 μL of the detection reagent should be added to 2.6 mL of deionized water, and 100, 200, 300, and 400 μL of MgCl_2_ salt solution with a concentration of 2×10^−3^ mol/L should be added to the above mixture. The resulting data from these two samples should then be processed to obtain Figure 9A.

**Procedure for Nuclear Magnetic Resonance Spectroscopy of Samples.** The structural changes of probe A before and after complexation with Mg^2+^ were analyzed using nuclear magnetic resonance hydrogen spectroscopy. The sample preparation method is outlined as follows: a quantity of 5 mg of solid probe A is dissolved in 0.6 mL of deuterated dimethyl sulfoxide. In the second step, 3 mg of solid probe A, together with 2 mg of MgCl_2_·6H_2_O dissolved in 0.6 mL of deuterated dimethyl sulfoxide, should be transferred from the above samples to NMR tubes for analysis by means of a pipette.

**Procedure for High-Resolution Liquid/Mass Spectrometry of Samples.** The structural characteristics of probe A complexed with Mg^2+^ were analyzed by high-resolution mass spectrometry. The experimental procedure entailed the preparation of a 2 mg sample of probe A, which was then dissolved in a 20 mL acetonitrile solution that contained 2 mg of MgCl_2_. Following this, the solution was filtered using a 0.22 μm microporous membrane. The filtered mixture was then transferred into a 2 mL injection bottle for sample analysis.

**Fluorescence Spectrophotometer Experimental Method**. The parameters of the two-dimensional fluorescence spectrum were set as follows: firstly, the instrument must be preheated for 30 min, with excitation and emission wavelengths both of 407 nm, a wavelength range of 420–650 nm, and excitation and emission slits of 10 nm. The detailed parameters for recording the three-dimensional fluorescence spectra were as follows: the excitation wavelength range was 200~600 nm and the emission wavelength range was 250~600 nm. The excitation slit and emission slit were both 5 nm, with a scanning speed of 1200 nm/min. The configuration method of the sample to be tested in the contour fluorescence spectrum involved the dissolution of MgCl_2_·6H_2_O, LiCl·6H_2_O, KCl, FeCl_3_·6H_2_O, NaCl, CsCl, ZnCl_2_, AlCl_3_, BaCl_2_·2H_2_O, and CaCl_2_ in deionized water to prepare a salt solution with a concentration of 2 × 10^−2^ mol/L. An amount of 100 μL of each solution was dissolved in 3 mL of deionized water. Subsequently, 10 μL of detection reagent should be added to each solution.

**Procedure for Fourier-Transform Infrared Spectroscopy Test.** The details are as follows. Wavelength range: 500–4000 cm^−1^, resolution: 4, and scanning times: 16. The sample should be prepared and 10 μL of the detection reagent should be dissolved in 3 mL of deionized water. Then, 10 μL of the detection reagent should be dissolved in 2.6 mL of deionized water, and 400 μL of the MgCl_2_ salt solution with a concentration of 2 × 10^−3^ mol/L should be added to the above mixture. Two samples should then be scanned and the resulting data processed to obtain Figure 2A.

**Theoretical calculation parameters.** In this work, density functional theory (DFT), molecular orbital theory, and the Fukui function from the DMol_3_ module were used for calculations. The molecular structure before electron density, electrostatic potential, and HOMO/LUMO calculations was optimized using the generalized gradient approximation (GGA) and BLPY functional. Before calculating the interaction energy, the structure was optimized using the generalized gradient approximation (GGA) and Perdew–Burke–Ernzerhof (PBE) model. The formula for calculating the interaction energy is E_int_ = E_A-B_ − (E_A_ + E_B_), where E_int_ represents the interaction energy between two molecules; E_A-B_ is the overall energy of the product; E_A_ is the energy of reactant A; and E_B_ is the energy of reactant B. The orbital ΔE = E (LUMO) − E (HOMO).

## 4. Conclusions

With the increasing demand for lithium carbonate in recent years, the rapid detection of the main Mg^2+^ impurity in lithium carbonate has received extensive attention. For many years, the detection of Mg^2+^ impurities has often been complicated, costly, and time-consuming. Therefore, it was challenging to provide timely guidance for the rapid refining and purification process of high-purity lithium carbonate. This study evaluates the solvation effect to design probe A, which shows a fluorescence response in saline solution, resulting in a water-soluble fluorescent probe method. First, the hydrophilic modification of polar groups is carried out to obtain fluorescent probe A. Its interaction energy with water molecules is 63 times that before modification and 1.8–20 times that of the other solvent molecules. The probe exhibits weak fluorescence in saline solution and shows good fluorescence stability due to the synergistic effect of solvation and the PET process.

Further analysis of the detection principle through microscopic interaction energy, electron density, and orbital gap changes revealed that the Mg^2+^ detection process is mainly influenced by water molecules and the PET blocking process. The fluorescence signal demonstrates a good linear relationship with Mg^2+^ concentration in the lithium carbonate solution, with a linear correlation coefficient of 0.994. The detection limit of this method is 6.06 μmol/L for Mg^2+^ detection, and the fluorescence response is completed within 3–10 s. At the same time, the detection process is unaffected by other ions. This study can efficiently solve the problem that the response of most fluorescent probes relies on the organic phase and is unsuitable for detecting Mg^2+^ impurities in lithium carbonate solutions, thus eliminating the need for sampling pretreatment. This method could lead to a breakthrough in overcoming technical difficulties of directly detecting Mg^2+^ impurities in high-purity lithium carbonate solutions in brine solutions and achieve online rapid detection.

## Data Availability

The original contributions presented in this study are included in the article/Supplementary Material. Further inquiries can be directed at the corresponding author.

## Supplementary Materials

The following supporting information can be downloaded at: www.mdpi.com/article/10.3390/molecules30040776/s1, Figure S1: Synthesis route of fluorescent probe A; Figure S2: Solid phase infrared absorption spectra of intermediate and final products in the reaction; Figure S3: Fluorescence spectra of detection reagents before and after identifying Mg^2+^ in different solvents; Figure S4: Fluorescence spectral changes of detection reagents for identifying different concentrations of Mg^2+^ in saline systems; Figure S5: The fluorescence intensity of the detection reagent changes with time in saline solution; Figure S6: The fluorescence intensity of the detection reagent varies with time under the condition of adding different amounts of triethylamine; Figure S7: At a triethylamine concentration of 0.333 mmol/L, the fluorescence intensity of the detection reagent changes with time; Figure S8: Fluorescence spectra of detection reagents for identifying different concentrations of Mg^2+^ in saline systems; Figure S9: Fluorescence microscopy images of hydrophilic modified substances in aqueous solution; Figure S10: Fluorescence microscopy images of detection reagents in aqueous solution; Figure S11: [Probe A+Mg^2+^] HRMS (High Resolution Mass Spectrometry); Figure S12: [Probe A+Mg^2+^]^1^H NMR (Nuclear Magnetic Resonance Spectroscopy of Hydrogen); Table S1: Calculation of the interaction energy between the probe and different solvent molecules; Table S2: Repetition of scans ten times to detect the peak fluorescence intensity of the reagent; Table S3: Orbital energy during the capture of Mg^2+^ by probe molecules.
