# Supplementary material for: Rapid Fluorescent Probe Detection of Magnesium Impurities in High-Purity Lithium Carbonate Brine Systems
Source: Molecules. 2025 Feb 7;30(4):776. doi: 10.3390/molecules30040776 (PMC11858617; doi:10.3390/molecules30040776)
Supplement: Supplementary file 1 [file molecules-30-00776-s001.zip › molecules-3387843-supplementary.pdf]

Supplementary Information

Rapid fluorescent probe detection of magnesium impurity in high-purity lithium carbonate brine systems

Yan Li[1], Huaigang Cheng[1,2]*, Yueyue He[1,3], Jing Zhao[1,2]

[1] Institute of Resources and Environmental Engineering, School of Environment and Resources, Shanxi University. Taiyuan 030006, China. E-mail: chenghg@sxu.edu.cn

[2] Salt Lake Chemical Engineering Research Complex, Qinghai University, Xining 810016, China; chenghg@sxu.edu.cnTable of contents

[3] Lvliang university, Lvliang 033000, China; 20241021@llu.edu.cn

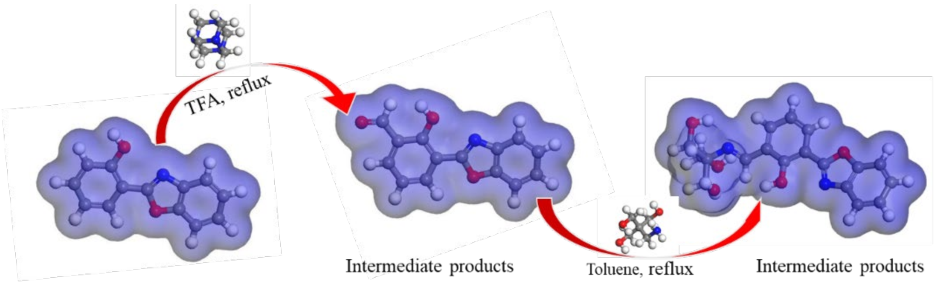

Figure S1 Synthesis route of fluorescent probe A

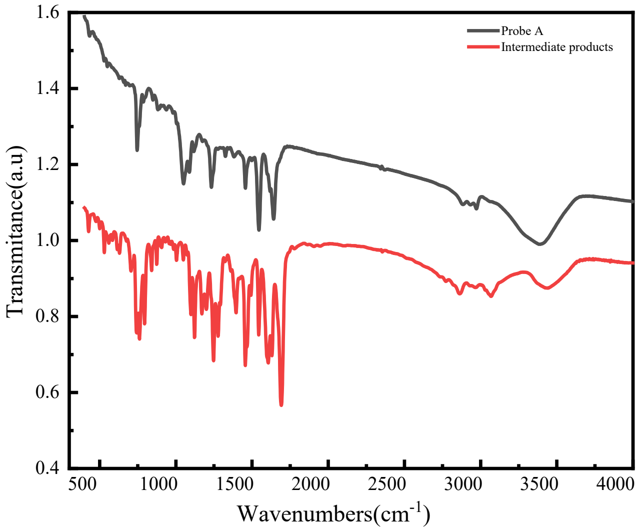

Figure S1 Solid phase infrared absorption spectra of intermediate and final products in the reaction

Table. S1 Calculation of the interaction energy between the probe and different solvent molecules

Material composition	E_{A-B}	$E_{A-Probe}$	$E_{B-solvent}$	E_{int}(Kcal/mol)
Pre modified substance+H_2O	-895.0865	-818.7034	-76.3827	-0.27108432
Probe+H_2O	-1257.83391	-1181.424142	-76.382757	-16.94942161
Probe-Mg^{2+}+H_2O	-1457.223462	-1380.417978	-76.382805	-265.2352993
Probe -ACN	-1314.085505	-1181.43891	-132.637584	-5.65449261
Probe -DMSO	-1734.390333	-1181.438238	-552.937128	-9.39194217
Probe -EtOH	-1336.361357	-1181.438896	-154.912186	-6.44766525
Probe -DMF	-1430.277189	-1181.436606	-248.821758	-11.81287575
Probe -MeOH	-1297.075856	-1181.4392	-115.63531	-0.84462846

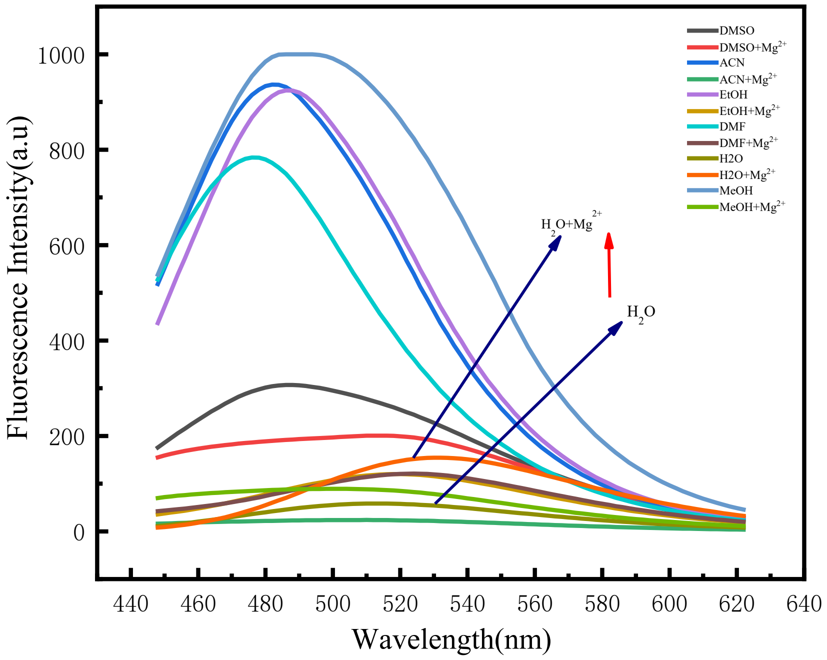

Figure S2 Fluorescence spectra of detection reagents before and after identifying Mg^{2+} in different solvents

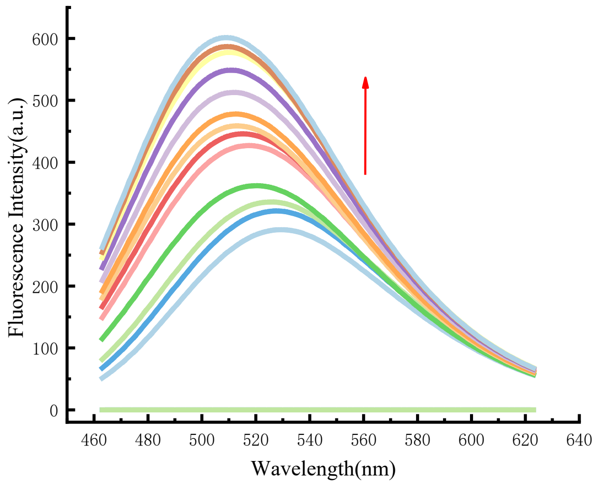

Figure S3 Fluorescence spectral changes of detection reagents for identifying different concentrations of Mg^{2+} in saline systems

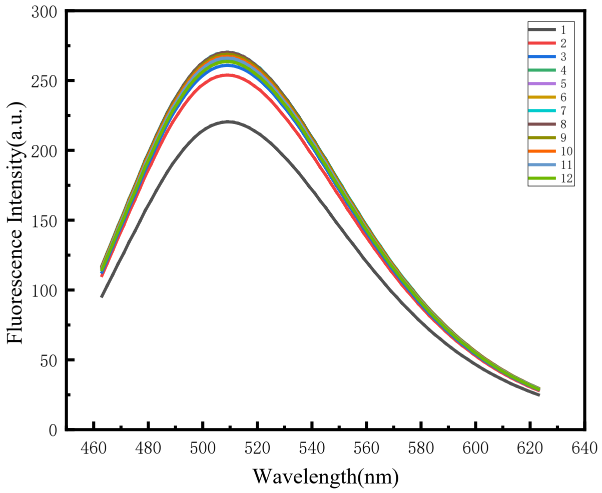

Figure S4 The fluorescence intensity of the detection reagent changes with time in saline solution.

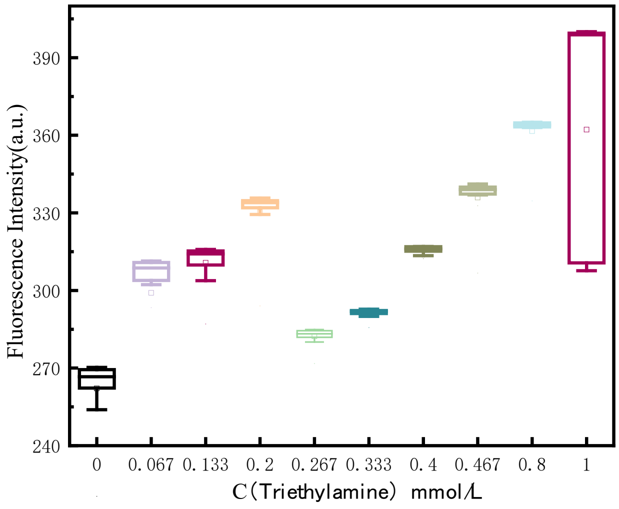

Figure S5 The fluorescence intensity of the detection reagent varies with time under the condition

of adding different amounts of triethylamine

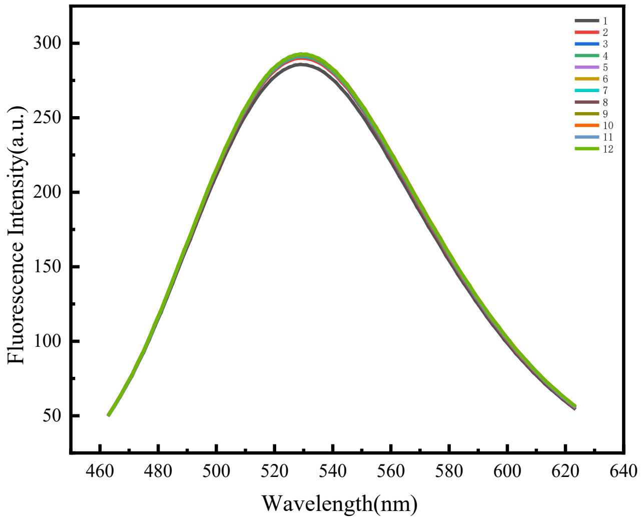

Figure S7 At a triethylamine concentration of 0.333 mmol/L, the fluorescence intensity of the detection reagent changes with time.

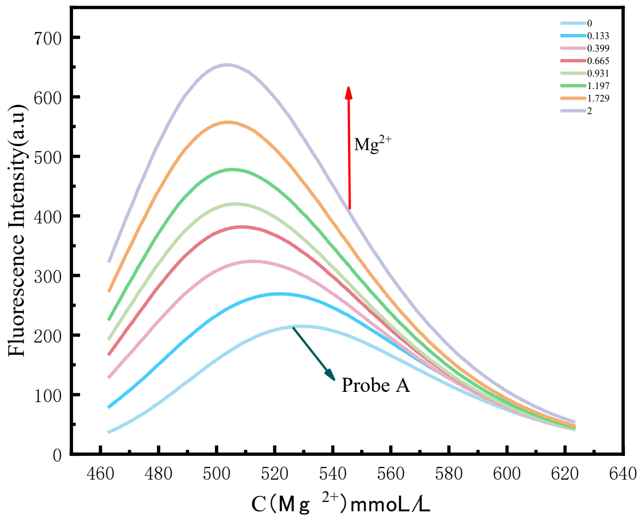

Figure S8 Fluorescence spectra of detection reagents for identifying different concentrations of Mg^{2+}in saline systems

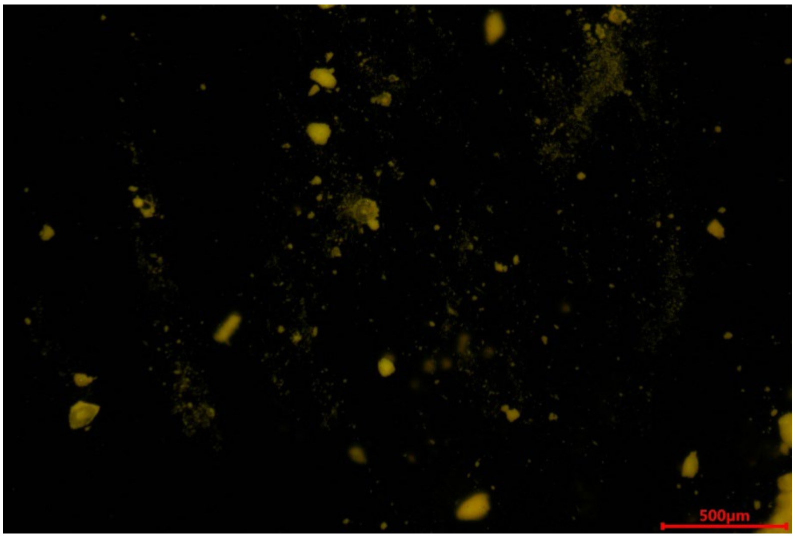

Figure S9 Fluorescence microscopy images of hydrophilic modified substances in aqueous solution

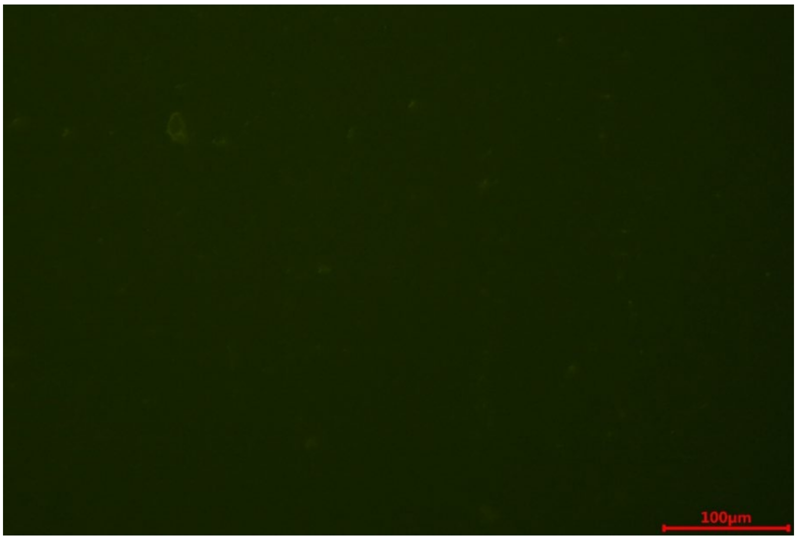

Figure S10 Fluorescence microscopy images of detection reagents in aqueous solution

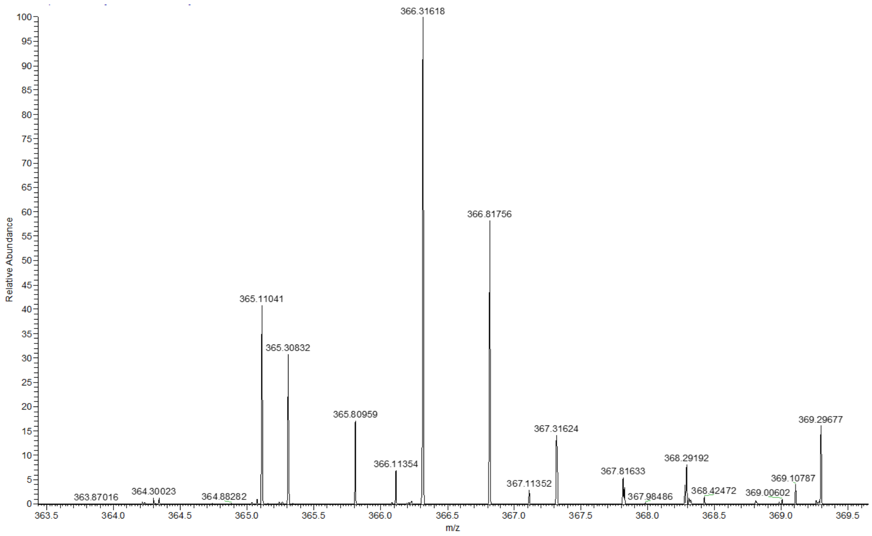

Figure S11 [Probe A+Mg^{2+}] HRMS（High Resolution Mass Spectrometry）

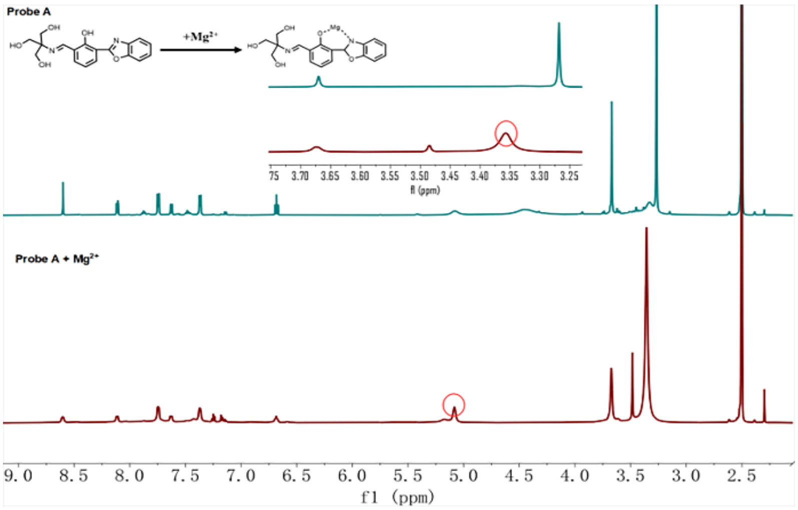

Figure S12 [Probe A+Mg^{2+}]^{1}H NMR（Nuclear Magnetic Resonance Spectroscopy of Hydrogen）

Details on the calculation of detection limit： DL = 3α/K；

The correlation between Mg^{2+} concentration and fluorescence intensity in lithium carbonate solution was calculated, as shown in Figure 5B red curve. The slope of the curve (K) was 264.088 L/mmol, scanning the detection of reagents in aqueous solution fluorescence intensity of 10 times (Table. S2) with a standard deviation of α = 0. 5335. The detection limit was calculated based on the International Union of Pure and Applied Chemistry (IUPAC) formula: DL = 3α/K. The calculated detection limit of Mg^{2+} impurity in high-purity lithium carbonate was 6.06 μmol/L

Table.S2 Repetition of scans ten times to detect the peak fluorescence intensity of the reagent

Number of scans	Fluorescence peak
1	237.2787
2	237.554
3	237.6006
4	238.5687
5	238.8385
6	238.6709
7	238.2948
8	238.1198
9	238.584
10	238.3081

Table. S3 Orbital energy during the capture of Mg^{2+} by probe molecules

Name of Chemical Substance	Energy（Ha）				ΔE=LUMO-HOMO	
	HOMO		LUMO		(Ha)	(eV)
Probe A	-0.191711Ha	-5.2166eV	-0.088184Ha	-2.3996eV	0.103527	2.8171
Probe A-Mg	-0.154202Ha	-4.196eV	-0.140135Ha	-3.8132eV	0.014067	0.3828
